# The challenge of durable brain control in patients with brain-only metastases from breast cancer

**DOI:** 10.1186/s40064-015-1384-x

**Published:** 2015-10-07

**Authors:** Carsten Nieder, Oliver Oehlke, Mandy Hintz, Anca L. Grosu

**Affiliations:** Department of Oncology and Palliative Medicine, Nordland Hospital, 8092 Bodø, Norway; Institute of Clinical Medicine, Faculty of Health Sciences, University of Tromsø, Tromsø, Norway; Department of Radiation Oncology, University Hospital Freiburg, Freiburg, Germany

**Keywords:** Breast cancer, Brain metastases, Radiotherapy, Prognostic factors, Progression-free survival

## Abstract

The vast majority of patients with brain metastases from breast cancer have extracranial metastases, e.g., in the liver, lungs or bones, with serious impact on prognosis. Limited research has been performed on patients with brain-only disease. We analyzed patterns of treatment, brain control and survival in uni- and multivariate analyses. All 25 patients with brain-only disease were treated with radiotherapy (whole-brain radiotherapy (WBRT) with or without stereotactic radiotherapy/radiosurgery (SRS) or surgical resection) and most patients with systemic treatment later during the disease trajectory. Only a minority of patients remained free from brain progression at 1 year after their initial therapy, regardless of initial treatment approach (median brain progression-free survival 6.2 months). However, overall survival was significantly better after initial surgical resection/SRS as compared to upfront WBRT (median 24.1 and 5.2 months, respectively). For all patients combined, median survival was 11.7 months (2-year survival rate 28 %). Several prognostic factors for shorter survival were identified in multivariate regression analysis: lower KPS, triple-negative tumor, coordination deficit, older age, lack of upfront surgical resection or SRS, and lack of endocrine or HER2-directed therapy after brain metastases treatment. Although durable brain control and long-term survival beyond 5 years could be achieved in a subset of patients (largely after successful salvage), progression of brain metastases during the first year after diagnosis was common. Prognosis was influenced by patient-, disease- and treatment-related factors.

## Background

Patients with newly diagnosed breast cancer often present with clinically localized stage I or II disease and have low rates of disease recurrence, especially in the brain. Arvold et al. (2012) reported a study of more than 1400 patients treated with breast-conserving therapy. Among patients who first developed extracranial metastases 34 % eventually developed brain metastases, whereas among women who did not develop extracranial metastases, <1 % developed brain metastases. Therefore, management of patients with brain-only metastases is less well studied, compared to the common scenario of both extra- and intracranial metastases (Lin et al. 2013).

Breast cancer subtype has been shown to correlate with risk of brain metastases. For example, Aversa et al. (2014) reported that patients with luminal tumors had the lowest risk. Ahn et al. (2013) found that time interval to diagnosis of brain metastases was shortest in the triple-negative group. In one of the largest studies with pooled data from 24 member institutions of the Japan Clinical Oncology Group, 1256 patients with brain metastases were included (Niikura et al. 2014). The median overall survival was 8.7 months. Patients with asymptomatic brain disease or HER2-positive/estrogen receptor-positive tumors had increased survival. In line with these results, Aversa et al. (2014) reported that median survival after diagnosis of brain metastases was shortest in patients with triple-negative tumors. Having isolated brain metastases predicted significantly reduced risk of death [hazard ratio (HR) 0.37, p < 0.004] in their study.

Since most studies included patients with and without extracranial metastases, and the results were dominated by the larger group of patients with extracranial metastases, we decided to perform a retrospective study in patients with brain-only metastases. Given that this situation is uncommon, we pooled data from two different institutions.

## Methods

All patients were females and treated for parenchymal brain metastases (not leptomeningeal disease) between 2003 and 2012, either in Freiburg or Bodø, and identified from the institutional electronic databases of the Departments of Radiation Oncology (ARIA, Varian Medical Systems, Inc., Palo Alto, CA, USA). Initially we screened 992 patients with brain metastases from different primary cancers. We included 21 breast cancer patients without extracranial metastases and 4 patients whose extracranial metastases were in complete remission after successful therapy. Regarding these 4 patients, brain metastases developed 6–13 months after complete remission of extracranial metastases. Only one patient relapsed in a previously known extracranial site during follow-up.

Overall, 17 % of patients with brain metastases from breast cancer had brain-only disease. First breast cancer diagnosis dated back to as long as 1986 in one of the patients. Staging consisted of computed tomography (CT; thorax, abdomen, pelvis) and isotope bone scan. If necessary to resolve uncertain findings, magnetic resonance imaging (MRI) and/or ultrasound were utilized. Screening for brain metastases in asymptomatic patients was not performed. Diagnosis was made by brain MRI and follow-up included further MRI every 3 months. Information about cognitive deficits and neurological symptoms was obtained from the physicians’ records. Prospective neurocognitive tests were not performed. Treatment consisted of whole-brain radiotherapy (WBRT), surgery, stereotactic radiotherapy/radiosurgery (SRS) or combinations thereof. Systemic therapy was at the discretion of the medical oncologists. At the time of analysis, 4 patients were alive (follow-up 29–70 months, median 48.5). Date of death was known in all other patients. Our definition of brain progression included the following events: new brain metastases or leptomeningeal enhancement, local relapse after surgical resection or after RT had resulted in complete remission, progression of previously treated lesions that never displayed complete remission (defined as an increase ≥25 % in at least one lesion, bidimensional measurement).

All analyses were performed with SPSS 22 (IBM, New York, USA). The Kaplan–Meier method was used to generate actuarial survival curves. These were compared with the log-rank test. Multivariate analysis of prognostic factors was performed with the Cox proportional hazards model (forward stepwise data selection method; inclusion if univariate p value <0.15; univariate analysis performed for all baseline parameters reported in the Results section). Age and Karnofsky performance status (KPS) were entered as continuous variables. A p value ≤0.05 was considered statistically significant. As a retrospective quality of care analysis, no approval from the Regional Committee for Medical and Health Research Ethics (REK Nord) was necessary. This research project was carried out according to our institutions’ guidelines and with permission to access the patients’ data.

## Results

Three patients (12 %) presented with brain metastases at first cancer diagnosis. These patients had untreated primary tumors. The median interval between first diagnosis and brain metastases was 29 months (range 0–292). Only five patients (20 %) had not received systemic therapy before diagnosis of brain metastases, including the three patients with synchronous presentation. Eighteen patients (72 %) were previously exposed to chemotherapy, 8 to endocrine therapy (32 %), and five to trastuzumab (20 %), typically in the adjuvant setting. The patient characteristics are shown in Table 1. Initial treatment was surgical resection in 10 patients (40 %), SRS in one patient (4 %) and WBRT in 14 patients (56 %). Including salvage therapy, all but one patient received WBRT during the disease trajectory. The most common regimen was 10 fractions of 3 Gy (48 %), followed by 14 fractions of 2.5 Gy (24 %). Salvage treatment included surgical resection (20 %), SRS (12 %), and fractionated stereotactic RT (20 %). After initial treatment for brain metastases 9 patients received chemotherapy (36 %), five endocrine treatment alone (20 %), and 3 trastuzumab (12 %). Overall, 10 patients remained without systemic therapy (40 %). If chemotherapy aimed at treatment of central nervous system relapse, the preferred options were capecitabine (n = 3) or temozolomide (n = 2).

**Table 1:** Patient characteristics, n = 25 (no male patients included)

Median age, range (years)	58, 37–82
Median KPS, range	80, 50–100
KPS 80–100 vs. 70 vs. <70 (%)	80, 16, 4
Single brain metastasis (%)	24
More than 3 brain metastases (%)	60
Infratentorial metastases only/supratentorial metastases only/both^a^ (%)	20, 40, 40
Controlled primary tumor (%)	88
Triple-negative tumor (%)	32
HER2 positive and ER/PR negative (%)	32
HER2 positive and ER or PR positive (%)	8
Headache (%)	28
Ataxia (%)	28
Dizziness (%)	32
Coordination deficit (%)	12
Visual disturbance (%)	12
Seizures (%)	16
Motor deficit (%)	16
Speech disturbance (%)	36
Cognitive deficit (%)	24
Asymptomatic (%)	8

*KPS* Karnofsky performance status, *ER* estrogen receptor, *PR* progesterone receptor

^a^Only one lesion was located in the brain stem

### Brain-progression-free survival (brain-PFS)

This endpoint was based on date of brain-progression detected on imaging studies (n = 11 events; only first progression was considered, not subsequent events in the same patient) or date of death if no progression was recorded (n = 10). Patients alive without progression were censored at last follow-up (n = 4). Median Brain-PFS was 6.2 months and one-year Brain-PFS 22 %. Table 2 shows disease characteristics associated with Brain-PFS. Four factors significantly predicted for better outcome in multivariate analysis: higher KPS, cerebellar metastases, lack of cognitive and coordination deficits.

**Table 2:** Univariate and multivariate analysis of predictive factors for brain-progression-free survival (PFS)

Parameter	Median brain-PFS, months	Univariate (log-rank test)	Multivariate p value^
1–3 vs. >3 brain metastases^a^	10.8 vs. 4.6	0.13	Not significant
Cerebellar vs. not	10.8 vs. 5.2	0.009	0.01
Cognitive deficit vs. none	3.1 vs. 10.8	0.02	0.02
Coordination deficit vs. none	4.6 vs. 6.9	0.06	0.01
KPS <80 vs. 80–100	2.1 vs. 8.9	0.13	0.009
Any systemic therapy vs. none (after local therapy for brain metastases)	9.8 vs. 4.6	0.02	Not significant

^Karnofsky performance status (KPS) and number of brain metastases were entered as continuous variables

^a^Different stratifications were tested, all with comparable results

### Time to brain progression

This endpoint was evaluated because not all patients had regular imaging follow-up. Events were registered as follows: brain-progression detected on imaging studies (n = 11; same criteria as outlined earlier), alive without progression (n = 4, censored at last imaging follow-up), not imaged after treatment for brain metastases (n = 5, censored at day 2 of treatment), imaging during initial follow-up only (n = 5, censored at last imaging follow-up). Median time to brain progression was 10.8 months and one-year freedom from progression 36 %. Four factors were associated with this endpoint (univariate): cerebellar metastases (p = 0.05), lack of cognitive and coordination deficits (p = 0.04 and 0.03, respectively), and systemic therapy after treatment of brain metastases (p = 0.06). However, multivariate analysis was unsuccessful because only 11 events were registered. All four patients who developed more than one progression in the brain eventually died from their brain metastases.

### Overall survival

**Fig. 1:**
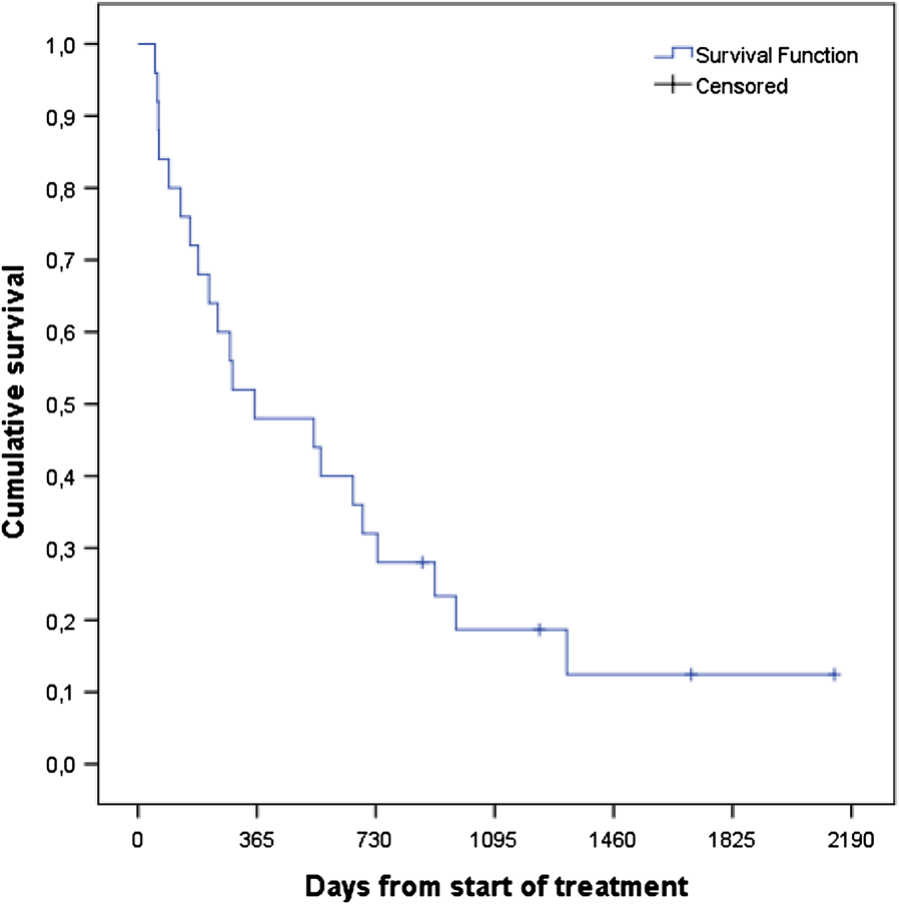
Kaplan–Meier estimate of overall survival in 25 patients with brain-only metastases from breast cancer (n = 4 censored observations)

**Fig. 2:**
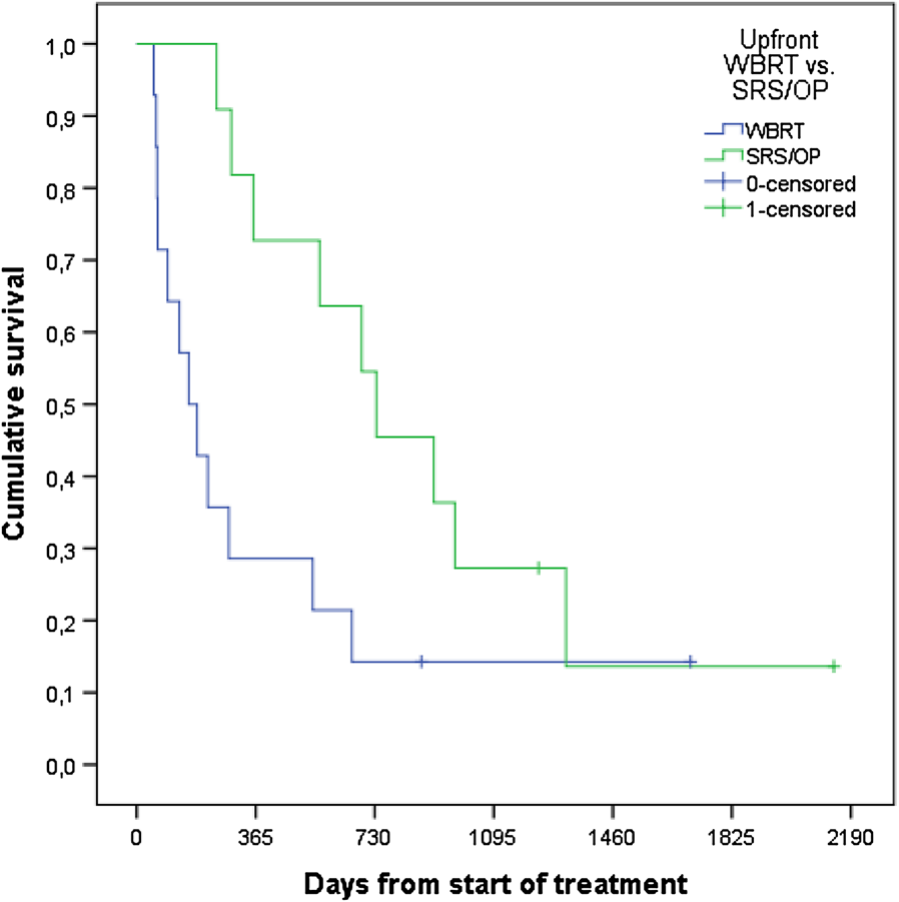
Kaplan–Meier estimates of overall survival in patients with brain-only metastases from breast cancer treated with upfront whole-brain radiotherapy vs. surgical resection or radiosurgery (n = 14 and 11, respectively). Median 5.2 vs. 24.1 months, p = 0.04 (log-rank test)

**Fig. 3:**
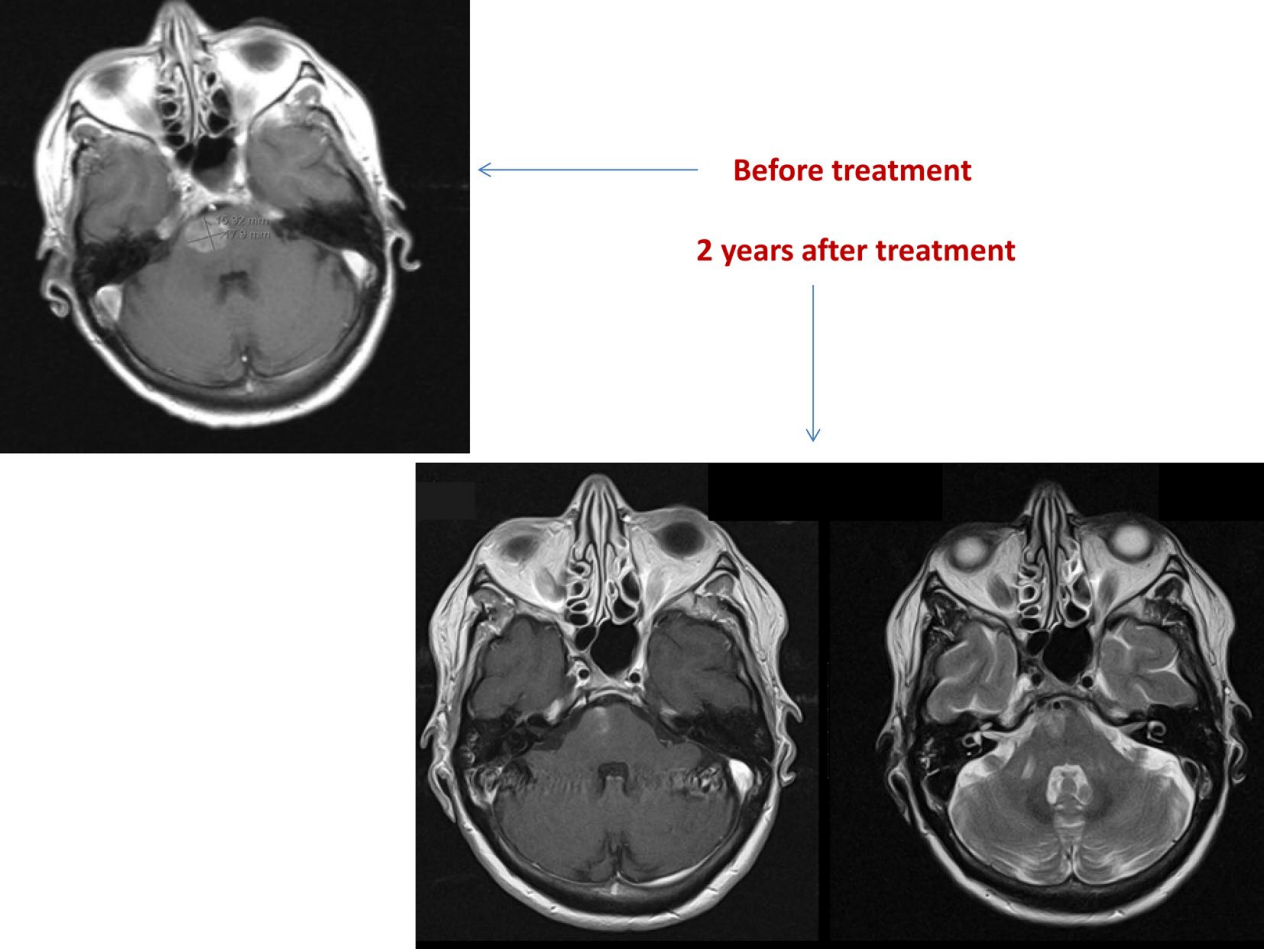
Magnetic resonance images before stereotactic fractionated radiotherapy in a 61-year old patient with solitary metastasis in the brain stem, HER2 positive disease, excellent performance status. The lesion received 7 fractions of 5 Gy prescribed to the 80 % isodose line. Two years later, radiation-induced changes developed, which resolved after treatment with steroids and pentoxifylline. The patient is alive without relapse

Median survival was 11.7 months, 1-year survival rate 48 %, and 2-year survival rate 28 % (Fig. 1). In the Cox model with baseline patient- and disease-related factors, three of these predicted for significantly shorter survival: lower KPS, p = 0.007, triple-negative tumor, p = 0.05, and coordination deficit, p = 0.005. If treatment-related variables also were included in the model, three additional factors emerged: older age, p = 0.04, lack of upfront surgical resection or SRS, p = 0.01, and lack of endocrine or HER2-directed therapy after brain metastases treatment, p = 0.01. Lack of endocrine or HER2-directed therapy after brain metastases treatment relates to the whole population irrespective of receptor status. Figure 2 shows the Kaplan–Meier curves for upfront WBRT vs. surgical resection/SRS. Median survival was 5.2 and 24.1 months, respectively. Figure 3 shows an example of the clinical course after successful radiotherapy.

## Discussion

The aim of the present retrospective analysis was to evaluate management approaches and outcomes in patients with breast cancer who developed isolated spread to the brain. In our database from two different institutions only 17 % of patients with brain metastases from breast cancer had brain-only disease. In contrast to a prospective trial, staging was not standardized. Rather routine clinical practice guided the use of different imaging modalities. Possibly, prospective imaging protocols might have detected small extracranial metastases in some of the patients included in our study. The low frequency of brain-only disease was unexpected and limited the statistical power of our analyses. In a multi-institutional study with 400 patients 35 % were free from extracranial metastases (Sperduto et al. 2012). However, detailed analyses of this subgroup were not performed.

It has long been recognized that presence of extracranial metastases is an important prognostic factor in the general population of patients with brain metastases (all primary tumors combined). For example, in the large study based on the Radiation Therapy Oncology Group (RTOG) database, median survival was significantly different, 4.8 vs. 7.1 months (Gaspar et al. 1997). Possibly, the impact is slightly less pronounced in patients with primary breast cancer, because the difference observed in the multi-institutional study mentioned above was beyond the limit of statistical significance (median 12.9 vs. 15.5 months) (Sperduto et al. 2012). The absolute difference was comparable (2.3 and 2.6 months, respectively). Regardless of statistical significance, the presence of extracranial disease is clinically relevant, because of its potential implications on organ function and choice of systemic therapy (Altundag et al. 2007; Bartsch et al. 2006, 2007).

In our study, three patients (12 %) presented with brain metastases at first cancer diagnosis. The median interval between first diagnosis and brain metastases was 29 months (range 0–292). We also included four patients whose extracranial metastases were in complete remission after successful therapy. Their outcomes were fully comparable to those observed in patients without preceding other metastases. For example, one patient was alive after 29 months of follow-up, whereas three had died after 9, 18 and 43 months, respectively. Treatment was individualized and consisted of WBRT, surgery, SRS or combinations thereof. Systemic therapy was frequently prescribed, e.g., continuation of trastuzumab in patients who developed brain-only metastases during adjuvant treatment or while in complete remission after eradication of extracranial metastases. A weakness of our study is the fact that course of extracranial disease after diagnosis of brain metastases was not known in most patients. However, 10 patients (40 %) remained without systemic therapy, suggesting that development of extracranial metastases is not inevitable. Due to the lack of complete information on long-term extracranial disease status and the fact that many patients received terminal care at places other than the authors’ institutions, causes of death could not be analyzed.

Berghoff et al. (2012) performed a retrospective study covering the time period 1990–2011 that will be discussed in greater detail. Out of 60 patients with brain-only recurrence, 37 % developed extracranial metastases during follow-up (median 7 months). The remaining 38 patients formed the final study cohort. Breast cancer subtype was not associated with brain-only metastatic behavior. Median survival was 11 months, as compared to 11.7 months in our study. These figures are within the lower range of the 95 % confidence interval of the multi-institutional series (10.1–19.8 months) (Sperduto et al. 2012). Berghoff et al. (2012) identified two significant prognostic factors, better KPS and single brain metastasis. Our results were somewhat different (KPS, triple-negative tumor, coordination deficit, age, surgical resection or SRS, and endocrine or HER2-directed therapy after brain metastases treatment), possibly as a result of the limited number of patients in both studies. With regard to our results it is important to realize that patients with triple-negative tumors are not eligible for endocrine or HER2-directed therapy. Niwinska et al. (2011) reported that patients with solitary brain metastasis (single, brain-only; n = 29) survived for a median of 20 months.

A large number of studies have investigated prognostic factors for survival in patients with brain metastases from breast cancer and several collaborative groups have developed prognostic scores (Sperduto et al. 2012; Nieder et al. 2009). A recent study by Subbiah et al. (2015) was based on the breast graded prognostic assessment (breast-GPA), comprising age, tumor subtype, and KPS. Data were retrospectively gathered from a prospectively maintained institutional database (1996–2013, n = 1552). The authors suggested a modified breast-GPA, which also includes number of brain metastases (>3 vs. ≤3). In our study, age, tumor subtype and KPS were statistically significant too. Number of metastases was not significant, but the treatment-related parameter surgical resection or SRS is related to the presence of more than 3 brain metastases.

The previously discussed study by Berghoff et al. (2012) did not analyze brain control. Our data suggest that durable brain control is difficult to achieve. Median Brain-PFS was 6.2 months and one-year Brain-PFS 22 %. Even after excluding death without documented brain-relapse as one of the events that influence the Kaplan–Meier curves, one-year freedom from progression was limited to 36 %. It is known from previous studies that upfront surgery/SRS without WBRT carries a considerable risk of distant brain progression, i.e. new lesions outside of the initially treated region(s) (Nieder et al. 2014). At the same time, upfront WBRT employs palliative radiation doses, which are not sufficient to permanently eradicate all visible metastases, in particular those with large volume (Nieder et al. 1998; Mahmoud-Ahmed et al. 2002). The dominant pattern of progression is growth of known lesions (Nieder et al. 1995). In principle, combination treatment (high doses to macroscopic tumor plus reduced doses to the other parts of the brain) might be beneficial (Rodrigues et al. 2013; Oehlke et al. 2015). However, no randomized trials have been performed that restricted inclusion to patients with brain-only disease. In mixed patient populations, no survival improvement was evident. Drugs that might reduce the risk of brain failure are not part of present clinical treatment algorithms. However, ongoing research efforts might eventually lead to more efficacious approaches (Kodack et al. 2015). With existing regimens, temporary responses can be obtained, which also might improve clinical symptoms in the context of palliation (Bachelot et al. 2013; Sutherland et al. 2010).

## Conclusions

It is presently unknown why selected patients with breast cancer develop brain-only metastatic disease, while most relapse in extracranial sites. Our data confirm previous studies that suggested a potential for long-term survival, also in patients who achieved complete remission of extracranial metastases after systemic treatment. Especially with upfront surgical resection/SRS median survival was 24.1 months. However, salvage treatment is often required. It is therefore important to repeatedly assess optimal local therapy and indication for systemic treatment in multidisciplinary expert panels.
